# A New Classification of Inferior Alveolar Nerve Repositioning Procedures for Dental Implant Placement

**DOI:** 10.3390/dj13060267

**Published:** 2025-06-16

**Authors:** Fares Kablan

**Affiliations:** 1 Department of Oral and Maxillofacial Surgery, Galilee College of Dental Sciences, Galilee Medical Center, Nahariya 2210001, Israel; kablanp1@gmail.com; 2 The Azrieli Faculty of Medicine, Bar Ilan University, Safad 1311502, Israel

**Keywords:** inferior alveolar nerve, atrophic mandible, dental implants, bone grafts

## Abstract

**Background**: Tooth loss significantly impacts the quality of life for adults. Inferior alveolar nerve (IAN) repositioning has garnered interest as a treatment for facilitating dental implant placement in the severely atrophic posterior mandible. However, there remains a need for standardization and classification of these techniques to improve outcomes. This study aims to propose a new clinical classification system for IAN repositioning procedures based on anatomical and procedural parameters. **Methods**: This study retrospectively analyzed preoperative radiographic records and surgical procedure documents over a 15-year period (2008–2023) for patients who underwent implant placement combined with IAN repositioning in the posterior atrophic mandible. Cases were classified into four categories according to bone availability, nerve location, and type of surgical intervention. **Results**: The study analyzed 142 edentulous posterior mandibles in 105 patients (77 women, 28 men; age range: 20–75). The cases were divided into four categories: Category 1 (58 patients, 78 sites), treated with one surgery; Category 2 (15 patients, 15 sites), treated in two stages; Category 3 (20 patients, 25 sites); and Category 4 (12 patients, 24 sites), with Categories 3 and 4 treated in a single surgery. Across all 132 sites, 411 dental implants were placed and restored with implant-supported fixed prostheses. **Conclusions**: This proposed classification provides a structured systematic framework for assessing and planning IAN repositioning procedures. It facilitates better diagnosis, treatment planning, and prediction of surgical stages in patients needing IAN repositioning for dental implant placement.

## 1. Introduction

Inferior alveolar nerve (IAN) mobilization and repositioning for dental implant placement was first reported by Jensen and Nock in 1987 [1]. Since then, it has become a widely accepted alternative to the use of short implants or bone grafts for dental implant placement in the posterior atrophic mandible. IAN repositioning can be performed using two main techniques: lateralization, which is performed posterior to the mental foramen; and transposition, which includes the mental foramen and sectioning of the incisive branch [2].

IAN repositioning offers several advantages, including reduced treatment time, decreased need for bone grafting, and the possibility of immediate dental implant placement during the same surgery. It also allows for bicortical implant anchorage and a favorable corono-radicular relationship [2,3]. In edentulous patients with severe mandibular atrophy, several treatment options are available for mandibular rehabilitation [4,5]; however, inferior alveolar nerve (IAN) repositioning may be the only viable approach for restoring the posterior edentulous region with a fixed implant-supported prosthesis [6].

Typically, patients requiring nerve repositioning are classified according to Cawood and Howell as having a posterior edentulous mandible of Class IV, V, or VI [7]. In clinical practice, the decision to proceed with IAN repositioning is based on a combination of anatomical and procedural factors. Based on my experience, the available bone for IAN repositioning is considered in six dimensions: the bone height over the nerve, bone width above the nerve, mandibular bone angulation, total mandibular height, total mandibular width, and location of the mandibular neurovascular bundle. However, the current literature lacks a standardized system to classify patients undergoing IAN repositioning based on these critical factors. Despite the increasing use of IAN repositioning in clinical settings, the literature lacks a standardized classification system that integrates these anatomical and procedural elements to guide clinical decision-making. While previous studies have discussed surgical techniques and associated complications, the absence of a structured classification system hinders the ability to compare outcomes, anticipate the number of surgical stages, and optimize preoperative planning. For example, Abayev and Juodzbalys (2015) systematically reviewed IAN lateralization and transposition, emphasizing the need for a more structured classification system to guide treatment decisions and predict potential risks. Their work highlighted significant variation in surgical approaches based on anatomical and clinical parameters [8]. Del Castillo Pardo de Vera et al. (2008) reported on the application of IAN repositioning in severely atrophic mandibles, emphasizing the challenges of treating severely atrophic mandibles and the need for a more precise and standardized approach in such cases [9]. Furthermore, Vetromilla et al. (2014) reviewed the complications associated with IAN repositioning, underscoring the importance of a classification system to predict and manage these risks effectively [10]. A follow-up study by Abayev and Juodzbalys (2015) focused on neurosensory complications [11], reinforcing the need for a classification that is not only anatomical, but also treatment-oriented.

This paper proposes a novel classification system for inferior alveolar nerve (IAN) repositioning, based on the available bone volume, anatomical location of the nerve, and type of surgical intervention required. By addressing current gaps in the literature, this classification aims to enhance surgical planning and the predictability of treatment sequences. The paper defines each category in detail, provides illustrative diagrams, and discusses their clinical implications.

## 2. Materials and Methods

A retrospective analysis was conducted on the preoperative radiographic records and surgical procedure documents of patients treated with implant placement in conjunction with inferior alveolar nerve (IAN) repositioning of the posterior atrophic mandible over a 15-year period from 2008 to 2023.

**Figure 1:**
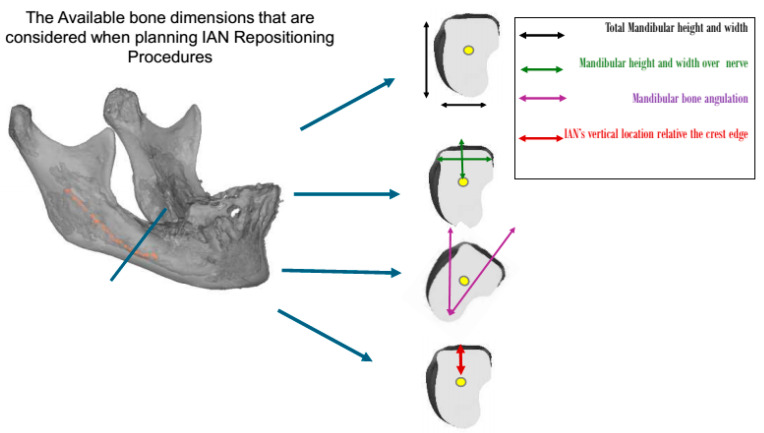
The residual ridge bone dimensions that should be evaluated when planning treatment for patients undergoing IAN repositioning procedures. Patient categorization was based on these dimensions.

This study was approved by the ethics committee of the Galilee Medical Center, Israel, under NHR-0130-24 (4 November 2024–4 November 2025). The cases were categorized, based on the available bone, nerve location (Figure 1), and surgical intervention, into four groups:

### 2.1. Category 1

**Figure 2:**
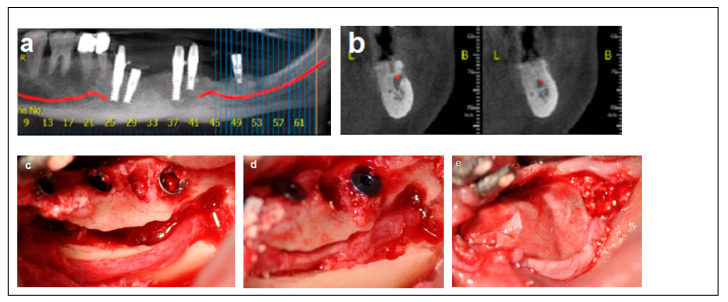

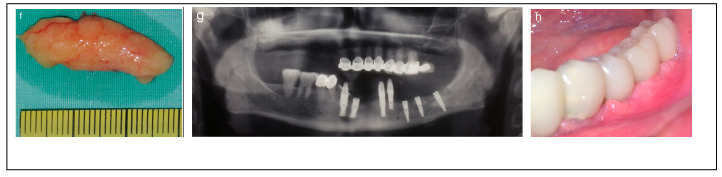
Illustrative case for Category 1. (**a**,**b**) Radiographic views showing a partially edentulous left posterior mandible with a straight ridge contour at the crest, good mandibular height, width and angulation (**c**) An intraoperative view of IAN transposition via a buccal window and placement of three implants. (**d**) An intraoperative view showing the repositioning of the IAN within the mandible. (**e**) The particulate bone substitute and resorbable membrane covering the surgical site. (**f**) The BFFG for primary closure. (**g**) Postoperative radiographic view. (**h**) Rehabilitation with a fixed prosthesis over the implants.

Criteria: Patients selected for the procedure met the following criteria: a mandibular height > 10 mm, straight mandibular bone or angulation < 15°, mandibular width > 6 mm, and bone height over the inferior alveolar nerve (IAN) between 3 mm and 6 mm. Treatment: The procedure was performed in a single-stage surgery, involving IAN mobilization and repositioning with immediate implant placement. A buccal bone window was created via ostectomy to access the nerve, which was carefully mobilized and repositioned within the prepared bone window. Dental implants (Spiral, Alpha-Bio Tec, Modi’in, Israel) with diameters of 3.75 mm and 4.2 mm, and lengths of 11.5, 13, and 16 mm, were placed in optimal positions to support a fixed prosthesis. Primary stability was achieved with an insertion torque of 30–35 Ncm. To enhance bone healing and prevent direct contact between the nerve and soft tissue, the buccal bone window was grafted with particulate bone (Raptos, Citagenix, Laval, QC, Canada) and covered with a resorbable membrane (Bio-Gide, Geistlich Pharma, Wolhusen, Switzerland). Additionally, a free buccal fat graft was utilized to augment the soft tissue and facilitate primary closure, ensuring improved wound healing and tissue adaptation (Figure 2).

### 2.2. Category 2

**Figure 3:**
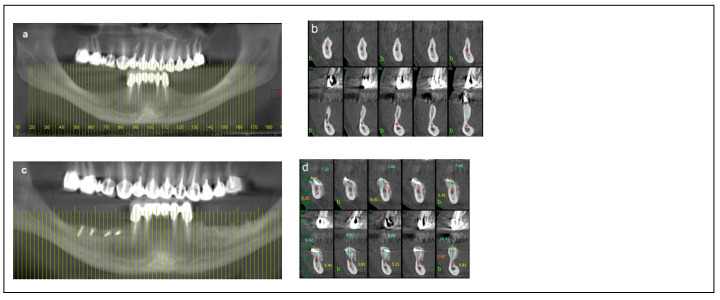

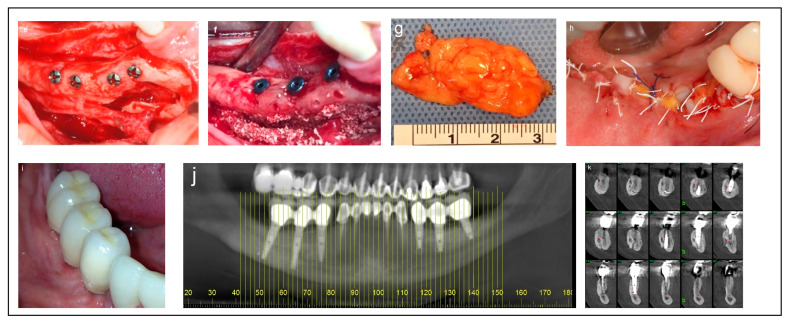
Illustrative case for Category 2. (**a**) A radiographic view of a partially edentulous right posterior mandible. (**b**) A CBCT view showing a narrow mandibular ridge with horizontal deficiency. (**c**,**d**) Radiographic views after bone grafting, demonstrating significant bone gain (**e**) An intraoperative view showing the bone graft before removing the fixation screws, alongside IAN transposition. (**f**) Intraoperative views depicting the placement of three implants and the repositioning of the nerve. (**g**,**h**) BFFG was used to enhance primary soft tissue closure. (**i**,**j**) Rehabilitation with a fixed prosthesis over the implants. (**k**) Cross-sectional CBCT views showing the implants and the new bone volume. (b, marked in yellow, indicates the buccal side).

Criteria: Patients in this category had a mandibular height between 6 mm and 10 mm, mandibular bone angulation > 15°, mandibular width < 3 mm, and bone height over the inferior alveolar nerve (IAN) between 3 mm and 6 mm. Treatment: The procedure was completed in two stages. The first stage involved bone augmentation, which could be horizontal (Division 1), vertical (Division 2), or combined (Division 3), depending on the specific anatomical deficiency. After a healing period of four months, the second stage was performed, including inferior alveolar nerve (IAN) transposition along with dental implant placement, as satisfactory prosthetic positioning could not be achieved through IAN repositioning alone. Dental implants (Spiral, Alpha-Bio Tec, Modi’in, Israel) with diameters of 3.3 mm and 3.75 mm, and lengths of 11.5 mm and 13 mm, were placed. Access to the nerve was obtained from the buccal aspect through a bone window, and the IAN was carefully mobilized and repositioned within the prepared buccal window. As in Category 1, the buccal bone window was grafted with particulate bone (Raptos, Citagenix, Laval, QC, Canada) and covered with a resorbable membrane (Bio-Gide, Geistlich Pharma, Wolhusen, Switzerland) to enhance bone healing and prevent direct contact between the nerve and soft tissue. Additionally, a free buccal fat graft was utilized to augment the soft tissue and facilitate primary closure. During implant placement, primary stability was achieved with an insertion torque of 30–35 Ncm (Figure 3).

### 2.3. Category 3

**Figure 4:**
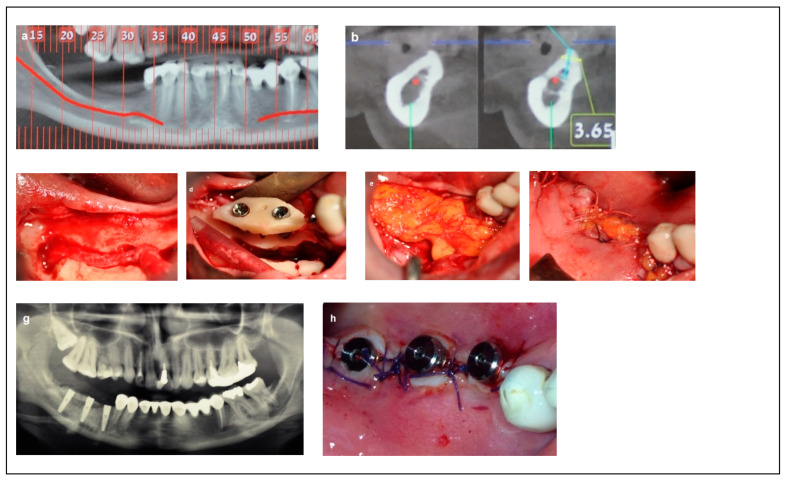
Illustrative case for category 3. (**a**,**b**) Radiographic views showing moderate mandibular bone dimensions. (**c**) IAN transposition via buccal window access. (**d**) Placement of two implants combined with an onlay bone block. (**e**,**f**); The use of a buccal fat free graft (BFFG) for primary closure of the grafted site. (**g**) Postoperative radiographic view. (**h**) The implant uncovering stage; note the thick soft tissue.

Criteria: Patients in this category had a mandibular height between 6 and 8 mm, mandibular bone angulation > 15°, mandibular width > 6 mm, and bone height over the inferior alveolar nerve (IAN) between 3 mm and 6 mm. Treatment: The procedure was completed in a single-stage surgery. The moderate bone dimensions in terms of height, width, and angulation allowed for IAN mobilization and repositioning, followed by the immediate placement of implants (Spiral, Alpha-Bio Tec, Modi’in, Israel) with diameters of 3.3 mm and 3.75 mm, and lengths of 11.5 mm and 13 mm. Access to the nerve was obtained from the buccal aspect through a bone window, and the IAN was carefully mobilized and repositioned within the prepared buccal window. Simultaneous bone augmentation was performed using either onlay bone blocks or guided bone regeneration (GBR) to enhance bone volume and support long-term implant stability. As in Category 1 and 2, the buccal bone window was grafted with particulate bone (Raptos, Citagenix, Laval, QC, Canada) and covered with a resorbable membrane (Bio-Gide, Geistlich Pharma, Wolhusen, Switzerland) to enhance bone healing and prevent direct contact between the nerve and soft tissue. Additionally, a free buccal fat graft was utilized to augment the soft tissue and facilitate primary closure. During implant placement, primary stability was achieved with an insertion torque of 30–35 Ncm (Figure 4).

### 2.4. Category 4

**Figure 5:**
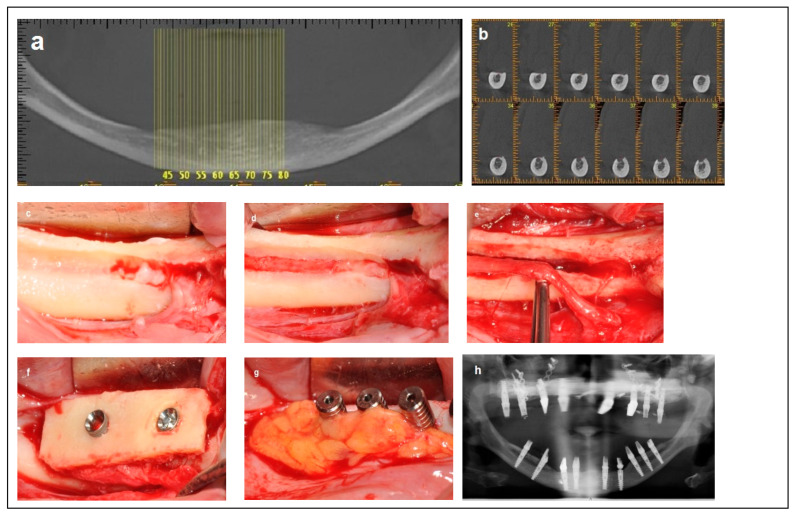
Illustrative case for Category 4. (**a**) Fully edentulous mandible with extreme atrophy. (**b**) A CBCT view showing a severely atrophied mandibular basal bone with a superficially located nerve. (**c**) An intraoperative view highlighting the superficial position of the mental nerve and the superior placement of the IAN at the crest. (**d**) Access to the IAN was gained from the superior portion of the crest. (**e**) Superior repositioning (Superioralization) of the IAN. (**f**) Implant placement through the onlay bone block, with repositioning of the IAN beneath the graft (Roofing technique). (**g**) Application of a BFFG at the recipient site to enhance primary soft tissue closure and augment soft tissue volume. (**h**) Postoperative radiographic view.

Criteria: Patients in this category had a mandibular height < 8 mm, mandibular bone angulation < 15°, mandibular width > 6 mm, and superficial location of the inferior alveolar nerve (IAN), with bone over the nerve ranging from 0 mm to 2 mm. Treatment: The procedure was completed in a single-stage surgery. Access to mobilize the IAN was achieved by removing the thin layer of residual bone covering the nerve on the superior side of the crest (Superioralization) of the IAN. During the same surgery, a calvarial split bone block was used to augment the mandibular height and enable the placement of longer implants. The bone block acted as a ‘roof’ (Roofing), with the inferior alveolar nerve (IAN) carefully repositioned beneath it. Particulate bone (Raptos, Citagenix, Laval, Canada) was used to fill the gaps and cover the repositioned nerve, and a resorbable membrane (Bio-Gide, Geistlich Pharma, Switzerland) was placed over the surgical site to prevent direct contact between the nerve and soft tissue, and to enhance bone healing and stability. Additionally, a free buccal fat graft was utilized to augment the soft tissue and facilitate primary closure. Primary stability was achieved during implant placement with an insertion torque of 30–35 Ncm. Implants (Spiral, Alpha-Bio Tec, Modi’in, Israel) with diameters of 3.3 mm and 3.75 mm, and lengths of 10 mm, 11.5 mm, and 13 mm, were placed. The bone blocks were stabilized by the implants themselves (Figure 5).

### 2.5. Summary Table

A comprehensive comparative table is presented below, highlighting and contrasting the defining features of all four categories. It offers a side-by-side comparison of key parameters, including the available bone height and width, the position of the inferior alveolar nerve, the recommended surgical technique, and the number of surgical stages involved (Table 1).

## 3. Results

Between 2008 and 2023, a total of 142 edentulous posterior mandibular sites in 105 patients (77 women, 28 men; age range: 20 to 75 years) were treated with IAN repositioning. A retrospective analysis of preoperative radiographic records and surgical procedure documentation led to the categorization of the patients into four groups:

Category 1: 58 patients (78 posterior mandibular sites) underwent a single surgery involving IAN repositioning and simultaneous implant placement, with a total of 210 implants placed.

Category 2: 15 patients (15 posterior mandibular sites) underwent a two-stage procedure. The first surgery involved bone augmentation, followed by a second surgery 4 to 5 months later, which included IAN repositioning and simultaneous implant placement. This group received 45 implants.

Category 3: 20 patients (25 posterior mandibular sites) underwent a single surgery incorporating IAN repositioning, simultaneous bone grafting, and implant placement, with a total of 84 implants placed.

Category 4: 12 patients (24 posterior mandibular sites) underwent a single surgery combining IAN repositioning, simultaneous bone grafting, and implant placement, receiving a total of 72 implants.

**Figure 6:**
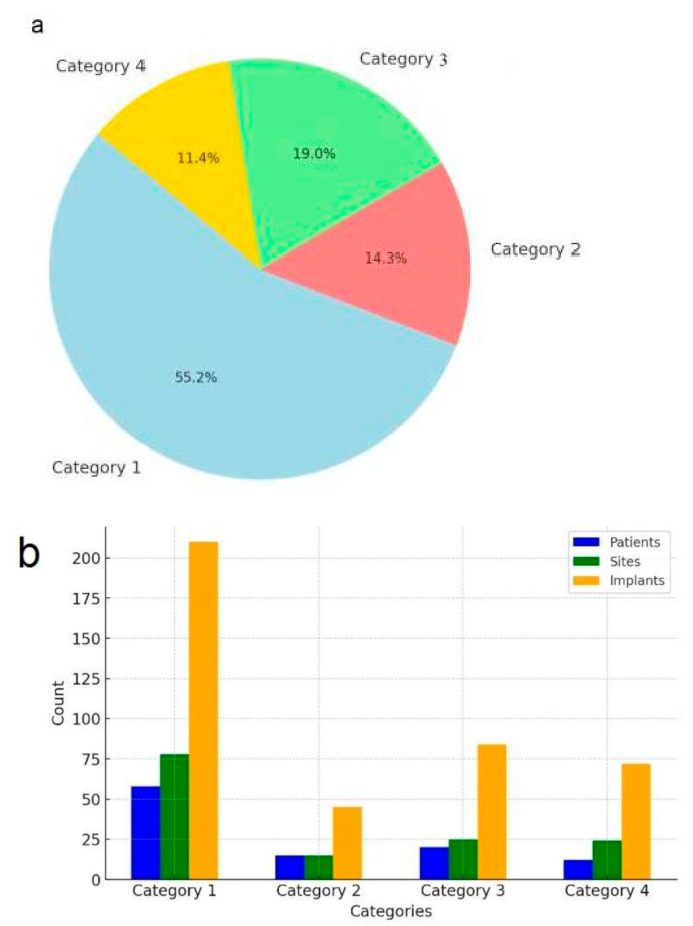
Distribution and summary of patients, treated sites, and implants by category. (**a**) Distribution of patients by category. (**b**) summary of patients, sites, and implants.

In total, 142 posterior mandibular sites treated with IAN repositioning received 411 dental implants, all of which were restored with implant-supported fixed prostheses (Table 2) (Figure 6).

## 4. Discussion

### 4.1. Anatomical, Surgical, and Biological Challenges in Implant Rehabilitation of Severely Atrophic Posterior Mandibles

Rehabilitating edentulous posterior mandibular regions with severe ridge atrophy using dental implants poses significant anatomical, surgical, and biological challenges. These factors complicate treatment for both the dental team and the patient. Many patients who rely on partial or complete dentures experience progressive instability, particularly affecting speech and mastication, leading them to seek fixed-prosthetic solutions [12,13]. Moreover, ongoing bone resorption in the posterior mandible may cause the inferior alveolar nerve to become more superficial, resulting in discomfort or pain during chewing [14]. In such cases, osseointegrated implants are generally considered the optimal solution for supporting fixed restorations. However, severe atrophy can limit the available bone height and increase the risk of inferior alveolar nerve (IAN) injury during implant placement. Recently, there has been growing interest in IAN repositioning as a viable approach for managing cases of mandibular atrophy, with several studies reporting satisfactory and predictable outcomes [15,16,17]. Various modifications to the IAN repositioning technique have been developed to reduce neurosensory complications and simplify the procedure for surgeons [18,19,20,21,22,23,24]. However, variability in anatomical conditions, surgical techniques, and clinical outcomes has highlighted the need for a standardized framework to guide patient selection and treatment planning. As noted in the systematic review by Abayev & Juodzbalys (2015), the choice between IAN lateralization and transposition depends on several factors, including the residual bone height, anatomical variations, and the surgeon’s experience. This comprehensive analysis remains one of the most detailed references available on IAN repositioning for implant rehabilitation [8].

Building on this foundation, I propose a stratified classification system for patients undergoing IAN repositioning. This system categorizes patients into four distinct groups, each characterized by specific clinical parameters that guide individualized treatment planning. Based on my clinical experience, key criteria for successful implant placement in the posterior mandible include evaluations of the residual ridge’s bone height, width, and angulation above the IAN. Additionally, the overall mandibular height, width, and angulation play a crucial role in determining the optimal implant position. The degree of mandibular bone resorption can also be inferred by assessing the depth of the IAN within the residual ridge; in cases of severe resorption, the IAN is located more superficially [25]. Another important consideration is the relationship between the residual ridge and the floor of the mouth. In cases of severe mandibular atrophy, this anatomical relationship can significantly impact plaque control [26], and should be integrated into the treatment plan. Categorizing patients based on these criteria provides a clearer understanding of the treatment process, the number of surgical interventions required, and whether grafting from a donor site surgery is necessary. While the Cawood and Howell classification categorizes ridge resorption by morphology, my system incorporates functional and surgical considerations. These include the position of the IAN within the residual ridge, the amount of bone over the nerve, and the feasibility of implant placement with or without bone grafting. This approach offers more practical value for clinicians planning IAN repositioning procedures, and provides procedural guidance beyond general morphology. The classification system include the following four categories. Category 1: Optimal bone dimensions for simultaneous IAN repositioning and implant placement. This category corresponds to Cawood and Howell Class V, and characterized by the following bone dimensions: 3 mm to 6 mm of bone above the inferior alveolar canal, a total mandibular height exceeding 10 mm, a width greater than 6 mm, and an angulation of less than 15 degrees. The ridge crest is typically located above the floor of the mouth. Patients in this category possess favorable anatomical conditions that allow for the placement of implants longer than 10 mm in a single surgery, enabling proper rehabilitation. Category 2: Bone augmentation required. Patients in this category correspond to Cawood and Howell Class IV or V, and present with increased mandibular angulation (greater than 15 degrees) or significant vertical and/or horizontal bone resorption that shifts the ridge crest lingually. In these cases, bone augmentation, either autogenous (harvested intraorally) or allogenic, is required to correct the ridge angulation or to increase the bone volume. After a healing period of 4 to 5 months, a second-stage surgery involving IAN mobilization and implant placement is performed. Implant survival rates in grafted bone blocks, whether allogenic or autogenous, are considered predictable [27]. This group also includes cases with ‘hourglass’ ridge morphology. A two-stage approach, as described by Proussaefs in 2005, involves vertical bone grafting followed by IAN transposition. He concluded that vertical ridge augmentation before IAN transposition is an effective strategy in cases with minimal bone height above the IAN canal [28].

Category 3: Moderate bone dimensions for simultaneous IAN mobilization and immediate implant placement. Patients in this category have moderate bone dimensions that allow for simultaneous IAN mobilization and immediate implant placement. Treatment is completed in a single surgery. Bone augmentation using either a block graft or guided bone regeneration (GBR) may be required to increase the bone volume around the implants or cover exposed implant surfaces. Additionally, simultaneous vertical augmentation can be performed to level the typically concave residual ridge, enhancing plaque control after rehabilitation. The mandibular width under the nerve canal should be more than 6 mm to adequately support the apical portion of the implants. In 2021, Ma et al., in a systematic review and meta-analysis, concluded that simultaneous implant placement with autogenous onlay bone grafting is an effective clinical approach, offering high implant survival rates and acceptable marginal bone loss in cases with horizontal bone deficiency [29]. Furthermore, the use of GBR techniques contributes to the stabilization of dental implants in patients with insufficient bone support [30]. Sethi et al. (2017) reported favorable outcomes using bone augmentation in combination with IAN repositioning for patients with insufficient bone height. In their study, implants were placed after the maturation of the grafts, and a high implant survival rate was observed after an average follow-up of 84.5 months [31].

A critical anatomical factor common to the first three categories is the presence of at least 3 mm of bone height above the IAN. This enables safe access to the nerve by removing a buccal window of cortical bone, parallel to the nerve’s path within the mandible. The IAN is then repositioned into this window, while maintaining a 2–3 mm band of bone on the crestal side to protect the repositioned nerve.

Category 4: Severe posterior mandibular atrophy. Patients in this category exhibit severe posterior mandibular atrophy, corresponding to extreme cases of Cawood and Howell Class VI, with significant reductions in mandibular height and width due to advanced resorption that involves the basal bone. This category also includes cases with an extramandibular course of the IAN, which the authors define as Class VII [25]. Such advanced atrophy is generally considered a contraindication for conventional IAN repositioning due to the profound anatomical changes involved [8]. In these patients, the IAN is located superficially, with only 0 to 2 mm of bone remaining above the nerve, and the mandible shows significant bone loss both vertically and horizontally. To address these challenges, the author introduced an innovative approach, referred to as IAN Superioralization, first presented as a clinical innovation at the Annual Meeting of the Academy of Osseointegration, 2016 (San Diego, CA, USA). This approach involves accessing the IAN via a superior route by removing the thin residual crestal bone covering the nerve. Once the IAN is mobilized, a calvarial bone block graft is placed to augment the mandibular height, enabling implant placement. The repositioned IAN is covered by the bone block graft, which acts as a protective roof, a concept referred to as IAN Roofing. This procedure can be performed in a single surgical stage, combining nerve repositioning, bone grafting, and implant placement. It is particularly indicated for partially edentulous patients (Kennedy Class I and II), with posterior mandibular atrophy where the anterior teeth remain intact, as well as for fully edentulous mandibles requiring full-arch implant-supported rehabilitation. According to data reported by the author in 2015, with follow-up periods ranging from 12 to 58 months, clinical outcomes were highly satisfactory in terms of stability, minimal bone loss, implant survival rates, and functional rehabilitation [32]. The use of calvarial bone grafts for augmenting atrophied ridges is well documented in the literature [33,34,35].

A soft tissue graft harvested from the buccal fat pad (BFFG) is routinely used in the case of Categories 2, 3, and 4 to augment soft tissue, improve ridge dimensions for better plaque control, and facilitate tension-free closure to prevent early wound dehiscence. Tension-free closure remains a critical factor influencing the success of bone grafting. To address this challenge, the author consistently employs a buccal fat pad-derived free fat graft (BFFG) for tension-free closure and simultaneous soft tissue augmentation at bone augmentation sites [36]. This technique effectively minimizes soft-tissue dehiscence while offering additional protection to the underlying bone graft. The author was the first to investigate and report the clinical and histologic healing process of a BFFG after maxillofacial surgery. BFFG has been shown to heal by rapid epithelialization of the exposed regions and simultaneous fibrosis of the graft that proceeds from immature fibrosis to full maturation after four months. The use of BFFGs in oral surgery is well documented [37,38,39,40,41].

### 4.2. Advantages

This classification system provides a structured framework that enhances clinical decision-making by categorizing patients based on anatomical parameters and treatment complexity. It offers several advantages. Firstly, it improves predictability in treatment planning; by categorizing patients into distinct groups, the classification allows clinicians to anticipate potential challenges, select the most appropriate surgical approach, and better manage patient expectations regarding procedural complexity, potential morbidity, and healing time. This structured methodology reduces uncertainties and improves preoperative preparation. In addition, it improves communication among clinicians. Standardized classifications facilitate clear communication between surgeons, prosthodontists, and referring dentists. By defining specific anatomical conditions and corresponding treatment protocols, the classification supports uniformity in case discussions, referrals, and multidisciplinary collaboration. Moreover, it enhances patient education and informed consent. Patients often struggle to understand complex surgical procedures and their implications. By assigning a classification category, clinicians can offer a clearer explanation of the expected treatment stages, the likelihood of additional grafting procedures, and the potential risks associated with each option. This transparency improves patient understanding, trust, and adherence to postoperative instructions. One of the primary concerns with IAN repositioning is the risk of neurosensory disturbances; by systematically categorizing cases based on nerve location, bone availability, and surgical complexity, clinicians can refine their technique selection to minimize nerve trauma. Additionally, tailoring treatment based on bone conditions can enhance implant stability and osseointegration and improve long-term prosthetic success. The lack of a widely accepted classification system for IAN repositioning has resulted in variability across published outcomes. By introducing a reproducible and clinically applicable classification, this study lays the groundwork for future comparative studies, long-term outcome evaluations, and refinements in surgical protocols. Researchers can use this framework to assess complications, success rates, and patient-reported outcomes across different categories, ultimately contributing to evidence-based advancements in the field.

To conduct this evaluation, a robust data collection system should be implanted, incorporating data from multiple sources, including patient records, clinical follow-ups, radiographic images, and patient feedback. Systematic analysis of these data points will enable validation, refinement, and optimization of the proposed classification system for IAN repositioning in dental implant placement, contributing to better clinical application and patient outcomes.

### 4.3. Strengths and Limitations of the Classification System

Strengths: Comprehensive and Structured: The system categorizes patients based on key anatomical features (bone dimensions, IAN position, ridge shape) and treatment needs (bone augmentation, grafting), making it more applicable to real-world clinical decision-making. Improved Predictability: By assessing the unique characteristics of the mandible, the classification provides a more predictable approach to surgical planning, allowing for tailored treatment protocols that reduce surgical complications. Standardization: This system can help to standardize the approach to IAN repositioning and implant rehabilitation, leading to improved communication among clinicians and better collaboration between oral surgeons, prosthodontists, and general practitioners. Patient-Centered: By improving patient education and providing a clearer understanding of the treatment process, the classification enhances patient satisfaction and aids in managing patient expectations.

Limitations: Complexity in Initial Assessment: The system requires a thorough preoperative evaluation, including radiographic imaging and detailed anatomical assessment, which may not be readily available in all clinical settings. This could increase the initial time investment for patient assessment. Limited Long-Term Data: While the classification system is based on clinical experience, long-term outcome data are needed to further validate its effectiveness across a broader patient population. Subjectivity in Categorization: In some cases, patients may fall into a gray area between two categories, which could lead to variability in treatment decisions or require a more nuanced interpretation of the anatomical parameters. Resource-Intensive: For more advanced categories (e.g., Category 4) requiring specialized grafts (such as calvarial bone grafting), the system may be resource-intensive and less feasible in some clinical environments, particularly those with limited access to advanced surgical techniques or materials. Complications, Success and Survival Rates, and Patient Feedback Not Included: This study does not include detailed data on complications, success rates, implant survival, or patient feedback, all of which are critical to fully understanding the outcomes of the proposed classification system. These aspects, along with demographic details of the patient population, will be addressed in future publications to provide a more comprehensive assessment of the system’s effectiveness and patient satisfaction over time. It is important to note that this classification is primarily descriptive, based on the anatomical features and surgical requirements observed in clinical practice. While the system offers a structured framework for treatment planning, it does not yet incorporate predictive elements related to individual patient outcomes, such as neural complications (e.g., paraesthesia, hypoesthesia), more severe issues like mandibular fractures, or long-term success rates [42,43]. Its primary purpose is to guide clinical decision-making, rather than to provide definitive predictions regarding surgical results or patient-specific prognoses.

Exclusion of Certain Anatomical Variations: The current system does not account for all anatomical variations that may significantly impact treatment decisions. For example, the presence of a bifid mandibular canal [44] or extremely dense bone (D1) [45] may represent contraindications for IAN repositioning, due to increased risk of nerve injury or technical difficulty. Furthermore, in cases where the IAN is located buccally or lingually, rather than centrally within the mandible, paraneural implant insertion may be a viable alternative to nerve mobilization, potentially avoiding the need for nerve repositioning [46].

Neurosensory Complications Not Included: This classification system is primarily based on anatomical and surgical characteristics, and does not currently incorporate data on neurosensory disturbance or other complications associated with inferior alveolar nerve (IAN) repositioning. Critical aspects such as the incidence, duration, and severity of transient or permanent nerve deficits, as well as other surgical morbidities, fall outside the scope of this framework. These outcomes are being evaluated separately, and will be addressed in future publications focusing on postoperative complications and long-term follow-up.

## 5. Conclusions

The proposed classification system is more than just a descriptive tool; it is a practical framework designed to optimize inferior alveolar nerve (IAN) repositioning procedures. By addressing the complexity of managing atrophic posterior mandibles, it enhances surgical planning, fosters better interdisciplinary collaboration, and improves patient education and understanding. This system supports a more standardized approach to treatment, which can reduce complications and improve clinical outcomes. As its adoption grows across clinical settings, the system’s validity and clinical utility will be further refined, offering valuable insights that could shape future guidelines for the surgical management of mandibular atrophy. Ultimately, this classification aims to bridge the gap between anatomical variability and surgical strategy, offering a robust foundation for better patient care and improved treatment consistency.

## Figures and Tables

**Table 1:** A summary table of the four clinical categories, their associated anatomical criteria, and the corresponding treatment approach.

Category	Title	Mandibular Height	Mandibular Width	Mandibular Angulation	Bone Over IAN	Treatment Protocol
Category 1	Straight-Ridge Favorable Bone	>10 mm	>6 mm	<15°	3–6 mm	One-stage IAN repositioning with immediate implants
Category 2	Deficient Ridge—Narrow/ Angulated	6–10 mm	<3 mm *	>15°	3–6 mm	Two-stage: augmentation followed by IAN repositioning + implants
Category 3	Moderate Bone Dimensions/Angulated	6–8 mm	>6 mm	>15°	3–6 mm	One-stage IAN repositioning + augmentation + implants
Category 4	Extreme Atrophy—Superficial Nerve	<8 mm	>6 mm	<15°	0–2 mm **	One-stage Superioralization of IAN + onlay bone graft + implants

* The main deference between Category 1 and Category 2 is the bone width. ** The main difference between Category 3 and Category 4 is the position of the inferior alveolar nerve within the mandible.

**Table 2:** Number of patients, treated sites, implants, and treatment stages by category.

Number of Implants	Number of Treatment Stages	Number of Sites	Number of Patients	Category
210	1	78	58	Category 1
45	2	15	15	Category 2
84	1	25	20	Category 3
72	1	24	12	Category 4
Total	105	142		411

## Data Availability

The required data can be obtained from the patients’ files.
